# Mucosal Healing and the Risk of Ischemic Heart Disease or Atrial Fibrillation in Patients with Celiac Disease; A Population-Based Study

**DOI:** 10.1371/journal.pone.0117529

**Published:** 2015-01-30

**Authors:** Benjamin Lebwohl, Louise Emilsson, Ole Fröbert, Andrew J. Einstein, Peter H. R. Green, Jonas F. Ludvigsson

**Affiliations:** 1 Celiac Disease Center, Department of Medicine, Columbia University College of Physicians and Surgeons, New York, New York, United States of America; 2 Department of Medical Epidemiology and Biostatistics, Karolinska University Hospital and Karolinska Institute, Stockholm, Swede0060045; 3 Primary care research unit, Vårdcentralen Värmlands Nysäter, Värmland County, and the Department of Medicine, Örebro University, Örebro, Sweden; 4 Department of Cardiology, Örebro University Hospital, Örebro, Sweden; 5 Division of Cardiology, Department of Medicine, and Department of Radiology, Columbia University College of Physicians and Surgeons and New York-Presbyterian Hospital, New York, New York, United States of America; 6 Department of Pediatrics, Örebro University Hospital, Örebro, Sweden; Johns Hopkins University SOM, UNITED STATES

## Abstract

**Background:**

Patients with celiac disease (CD), characterized histologically by villous atrophy (VA) of the small intestine, have an increased risk of ischemic heart disease (IHD) and atrial fibrillation (AF), risks that persist for years after commencing the gluten-free diet. It is unknown whether persistent VA on follow-up biopsy, rather than mucosal healing, affects the risk of IHD or AF.

**Methods:**

We identified patients with histologic evidence of CD diagnosed at all 28 pathology departments in Sweden. Among patients who underwent a follow-up small intestinal biopsy, we compared patients with persistent VA to those who showed histologic improvement, with regard to the development of IHD (angina pectoris or myocardial infarction) or AF.

**Results:**

Among patients with CD and a follow-up biopsy (n = 7,440), the median age at follow-up biopsy was 25 years, with 1,063 (14%) patients who were ≥60 years at the time of follow-up biopsy. Some 196 patients developed IHD and 205 patients developed AF. After adjusting for age, gender, duration of CD, calendar period, and educational attainment, there was no significant effect of persistent VA on IHD (adjusted HR 0.97; 95%CI 0.73–1.30). Adjusting for diabetes had a negligible effect (adjusted HR 0.98; 95%CI 0.73–1.31). There was no significant association between persistent VA and the risk of AF (adjusted HR 0.98; 95%CI 0.74–1.30).

**Conclusions:**

In this population-based study of patients with CD, persistent VA on follow-up biopsy was not associated with an increased risk of IHD or AF. Failed mucosal healing does not influence the risk of these cardiac events.

## Introduction

Celiac disease (CD) is an immune-based disorder characterized by small intestinal inflammation and villous atrophy (VA) that is triggered by the ingestion of gluten in genetically-susceptible individuals.[1] Now present in approximately 1% of the population of the United States, patients with CD may present with a myriad of symptoms.[2,3] Patients may exhibit diarrhea and malabsorption, but extra-intestinal manifestations are manifold and can include fatigue, metabolic bone disease, neuropsychiatric symptoms, and reproductive disorders.[1] Patients with CD have an increased risk of death due to cardiovascular causes, a risk that persists (though is attenuated) in the years following the diagnosis of CD and the commencement of a gluten-free diet.[4] Cardiovascular diseases that have been found to be associated with CD include atrial fibrillation (AF), [5] cerebrovascular events, [6] cardiomyopathy, [7] and ischemic heart disease (IHD).[8] Given that the IHD is the leading cause of death in the world, [9] and that AF is a significant contributor to cerebrovascular disease, [10] the increased risks of IHD and AF are important drivers of overall mortality risk among patients with CD.[4]

Patients with CD who develop IHD have a risk-factor profile that is distinct from patients without CD who develop IHD. [11] The risk of IHD in patients with CD may be conferred by a chronic inflammatory state.[11,12] This distinct risk is invoked because CD patients with IHD have a lower mean body mass index, lower total cholesterol, and lower rates of smoking than their non-CD counterparts with IHD. While similar risk factor analyses have not been performed in patients with CD who develop AF, the latter condition is associated with elevated inflammatory markers as well.[13]

Follow-up biopsy has emerged as a potentially important approach to risk-stratify CD patients with regard to long-term prognosis. Rates of persistent VA are variable in the literature (occurring in at least 30% of patients in most series), [14–19] but persistent VA is inversely correlated with adherence to the gluten-free diet.[16,18,19] While persistent VA does not impact overall mortality or mortality due to cardiovascular disease, [20] there have been no studies investigating a link between persistent VA and the *incidence* of IHD and AF, two cardiac conditions associated with CD. Persistent VA may be a marker of disease severity and ongoing inflammation, which may in turn impact the risk of cardiovascular disease. The finding of persistent VA is clinically relevant, as it can reflect gluten exposure and may motivate patients to improve dietary adherence.[14] Persistent VA has been shown to be associated with an increased risk of lymphoproliferative malignancy[21] and hip fracture, [22] two outcomes that are more common in patients with CD than among the general population. It is unknown whether findings from follow-up histology predict the incidence of IHD or AF in these patients.

We therefore aimed to determine whether the results of follow-up biopsy among patients with CD influence the risk of developing IHD or AF.

## Methods

### Identification of patients with CD

During the years 2006–2008 we queried all (n = 28) pathology departments in Sweden for reports of VA, as identified by SnoMed codes. Details regarding this population-based database have been published previously.[4,23] In brief, VA was identified by Swedish pathologists and a prior validation study demonstrated that, among patients identified via these histology codes, a clinical diagnosis of CD was present in 95% of patients; alternative/comorbid diagnoses were rare (with inflammatory bowel disease, the most common comorbidity, present in 0.3% of 1,534 manually reviewed patient records).[23]

The patients in this analysis are those with CD who underwent more than one duodenal biopsy; they therefore represent a subset of all CD patients, who were included in our earlier studies that found an increased risk of IHD and AF in CD.[5,8] We identified those CD patients who underwent follow-up biopsy between 6 months and 5 years after initial CD diagnosis. Those patients who had a modified Marsh histopathology score[24,25] of 3 were classified as having persistent VA, while those with a less severe score were classified as healed.

### Identification of cases of IHD

Data on IHD were obtained from the Swedish Patient Registry containing both inpatient data (since 1964, and nationwide since 1987) and hospital-based outpatient data (since 2001); data for fatal MI were obtained from the Swedish Cause of Death Registry.[26] The composite outcome of IHD consisted of either inpatient visits associated with myocardial infarction (MI) or angina pectoris, or death from MI. We used the same international classification of disease (ICD) codes as in our previous paper on IHD (ICD 10: I20–22 and corresponding ICD7–9 codes).[8]

### Statistical considerations

We used Cox proportional hazard models to compare the risk of risk of IHD among those with persistent VA versus those with mucosal healing on follow-up biopsy; we report risk estimates as hazard ratios (HR) and corresponding 95% confidence intervals (CI). Follow-up time began on the day of the follow-up biopsy and ended on the date of death, emigration, or development of IHD, or December 31, 2009, whichever occurred first. In this analysis we chose *a priori* the following covariates in the model: age at follow-up biopsy, gender, duration of CD at the time of follow-up biopsy, calendar period of follow-up biopsy, and educational attainment. In the case of children, the highest educational attainment of either parent was substituted. Because the risk of sequelae in CD (including mortality due to cardiovascular causes) changes over time, [4] we repeated this analysis, recalculating the association between persistent villous atrophy and the development of IHD during the following 3 pre-specified time strata: <1 year, 1–5 years, and >5 years after the patient’s follow-up biopsy. We then performed stratified analysis by age, gender, and calendar period of follow-up biopsy. In a post-hoc power analysis using an alpha of 0.05, we had 80% power to detect an association between persistent VA and IHD at a relative risk of 1.48.

### Sensitivity Analyses

We performed several additional analyses to test the robustness of our results. We repeated the primary analysis, now treating age as a continuous (rather than a categorical) covariate, and then using age as the time scale. So as to determine whether duration of VA was influencing the main association, we repeated the analysis, removing duration of CD from the multivariate model. Although diabetes mellitus has not been tested with regard to a potential impact on mucosal healing, because of its strong association with IHD we repeated the primary analysis, now including the presence of diabetes mellitus (type 1 or 2) as a covariate.

Persistent VA may be a consequence of longstanding untreated CD, i.e. prolonged gluten exposure prior to the biopsy diagnosis of CD. It is unknown whether such longstanding untreated CD could impact the development of IHD prior to CD diagnosis. For this reason, we subsequently sought to determine whether a prior diagnosis of IHD was associated with persistent VA among patients who were subsequently diagnosed with CD and then underwent follow-up biopsy. In this subsequent analysis, we assessed for this association using multiple logistic regression, adjusting for the same covariates as those used in our Cox model for the primary analysis.

### Atrial fibrillation

CD has been previously shown to increase the risk of subsequent development of AF.[5] So as to determine whether the development of AF influenced the results of our primary analysis, we tested for an association between persistent VA and the subsequent development of AF. For this analysis, we used the following diagnosis codes to identify inpatients and outpatients with AF: ICD7: 433.12; ICD8: 427.92; ICD9: 427D; ICD10: I48. We excluded all patients with a diagnosis of AF preceding the date of follow-up biopsy, and our adjusted risk estimates used the same covariates as those used for the primary analysis.

We used SAS version 9.3 (Cary, NC) for all statistical analyses. All reported p values are two-sided. This study was approved by the Research Ethics Committee of the Karolinska Institute on June 14th, 2006.

## Results

### Characteristics of patients who underwent follow-up biopsy

We identified 9,725 patients with CD who underwent a follow-up biopsy. Of these, 2,077 underwent a follow-up biopsy outside of the time frame of 6 months to 5 years after initial CD diagnosis. An additional 208 patients were excluded due to the development of IHD prior to their follow-up biopsy. The remaining 7,440 patients with CD who underwent follow-up biopsy were included for further analysis.

As has been reported previously, [20] those patients who underwent follow-up biopsy were slightly younger than those who did not (mean age at CD diagnosis 28.4 versus 33.4 years, p<0.0001). In our prior study examining the overall risk of IHD patients with CD, we matched each CD patient with up to 5 controls via Sweden’s Total Population Register, using the following matching parameters: age, gender, county, and calendar year.[8] In this subset of patients with CD who underwent follow-up biopsy, the overall risk of developing IHD as compared to these previously matched controls and stratified by age, gender, and year, is shown in S1 Table. The overall risk of IHD in these patients with CD compared to controls did not reach statistical significance (HR 1.09; 95%CI 0.95–1.25). This point estimate is lower than (though its confidence interval includes) the risk estimate previously reported for all patients with CD, regardless of whether a follow-up biopsy was performed (HR 1.19).[8]

Among those who had a follow-up biopsy during the pre-specified time period, 64% were female (Table 1). Some 45% of these patients had their follow-up biopsy performed between one and two years after their initial CD diagnosis. In the majority of patients (52%) follow-up biopsy was performed after the year 2000. Among those who had a follow-up biopsy, 43% exhibited persistent VA. We have previously reported that factors associated with persistent VA include older age, male gender, and low educational attainment.[27]

**Table 1:** Characteristics of the patient cohort with CD and follow-up biopsies.

Characteristic	Number (%)
Age at diagnosis of CD (years)	
0–19	3,407 (46)
20–39	1,332 (18)
40–59	1,638 (22)
≥60	1,063 (14)
Male	2,690 (36)
Female	4,750 (64)
Diabetes	
Yes	260 (3.5)
No	7,180 (96.5)
Interval between diagnosis and follow-up biopsy	
6 months–1 year	1,950 (26)
Between 1 and 2 years	3,352 (45)
2–5 years	2,138 (29)
Calendar period of *follow-up biopsy*	
≤1989	722 (10)
1990–1999	2,834 (38)
≥2000	3,884 (52)
Second biopsy result	
Mucosal healing	4,242 (57)
Persistent villous atrophy	3,198 (43)
Developed IHD during follow-up:*	196 (2.6))
Angina pectoris	111 (1.5)
Unstable angina pectoris	30 (0.4)
MI	176 (2.4)
Fatal MI	37 (0.5
Developed AF during follow-up:†	205 (2.7)

(Excludes patients with a diagnosis of IHD prior to follow-up biopsy, n = 208).

CD, celiac disease

IHD. Ischemic heart disease

MI, Myocardial infarction

*The sum of IHD subtypes is greater than the total number of patients with IHD because of patients who had more than one type of IHD event

†Among patients without a history of AF at the time of follow-up biopsy (n = 7,530).

### Risk of IHD

The median observation time after CD diagnosis was 10.6 years (IQR 7.1–16.0 years) and the median observation time after follow-up biopsy was 8.9 years (IQR 5.5–14.1 years). During this period, 196 patients (2.6%) developed IHD. Among patients with CD who had a follow-up biopsy, the overall incidence of IHD was 257 per 100,000 person-years. This rate was greater among those with persistent VA (298 per 100,000 person-years) as compared to those with mucosal healing (214 per 100,000 person-years), yielding a trend towards a positive unadjusted association (HR 1.30; 95%CI 0.97–1.73, p = 0.08).

After adjusting for age at follow-up biopsy, gender, calendar period of follow-up biopsy, degree of educational attainment, and duration of CD, there was no significant difference in risk of IHD between those with mucosal healing and those with VA (HR 0.97; 95%CI 0.73–1.30, p = 0.85). The primary confounding influence was age; adjusting for age alone resulted in the HR moving from 1.30 down to 1.07. In this model, factors associated with an increased risk of IHD included male sex (HR 2.08; 95%CI 1.56–2.78, p<0.0001), increased age (HR per year 1.09; 95%CI 1.08–1.10, p<0.0001), and lower degree of educational attainment (HR for college/university versus <2 years of high school 0.54; 95%CI 0.33–0.87, p = 0.01).

When stratifying risk of IHD over time after follow up biopsy (Table 2), in none of the pre-specified time strata did this association meet statistical significance. A test to determine whether the association between persistent VA and the development of IHD differs over time showed no significant interaction between persistent VA and time after follow-up biopsy with regard to the risk of IHD (p = 0.20).

**Table 2:** Association of persistent villous atrophy with IHD overall, and stratified by time after follow-up biopsy.

Stratum	Number of events	Incidence per 100,000 PY	Adjusted† HR (95% CI)	p value
Overall		257		
Mucosal healing	80	214	1.0	
Persistent villous atrophy	116	298	0.97 (0.73–1.30)	0.85
<1 year		189		
Mucosal healing	3	71	1.0	
Persistent villous atrophy	11	346	2.97 (0.83–10.7)	0.09
1–5 years		186		
Mucosal healing	24	156	1.0	
Persistent villous atrophy	27	224	0.90 (0.52–1.56)	0.69
>5 years		316		
Mucosal healing	53	299	1.0	
Persistent villous atrophy	78	329	0.90 (0.63–1.29)	0.57

HR, Hazard Ratio

†Adjusted for patient age at follow-up biopsy, gender, calendar period of follow-up biopsy, education, and duration of celiac disease at the time of follow-up biopsy

There was no observed association between persistent VA and the development of IHD when stratifying by gender, age, and calendar year after follow-up biopsy (Table 3). When restricting the population to adults (age ≥20 years), the incidence of IHD was 534 per 100,000 person years of observation, and there was no association between persistent VA and risk of IHD (HR 0.97; 95%CI 0.73–1.30, p = 0.85) Neither was there a significant association between persistent VA and IHD subtypes, including angina pectoris, unstable angina pectoris, myocardial infarction, and fatal myocardial infarction (Table 4). The association between persistent VA and IHD remained non-significant when repeating the analysis, now with age treated as a continuous covariate (HR 0.95; 95%CI 0.71–1.27, p = 0.72), when age was used as the time scale (HR 0.95; 95%CI 0.71–1.27, p = 0.12) and when excluding the duration of CD from the model (HR 0.98; 95%CI 0.74–1.31, p = 0.90). The relationship similarly was null when including the presence of diabetes mellitus in the multivariate model (HR 0.98; 95%CI 0.73–1.31, p = 0.88). The positive association between diabetes and IHD in this population did not meet statistical significance (HR 1.79; 95%CI 0.91–3.52, p = 0.09).

**Table 3:** Association of persistent villous atrophy with IHD, stratified by gender, age, and year of CD diagnosis.

Stratum	Number of events	Incidence per 100,000 PY	Adjusted† HR (95% CI)	p value
Gender				
Male		380		
Mucosal healing	41	307	1.0	
Persistent villous atrophy	63	450	0.94 (0.63–1.41)	0.76
Female		188		
Mucosal healing	39	163	1.0	
Persistent villous atrophy	53	213	1.07 (0.71–1.64)	0.74
Age at follow-up biopsy (years)				
<20		0		
Mucosal healing	0	0	1.0	
Persistent villous atrophy	0	0	—	—
20–39		71		
Mucosal healing	3	41	1.0	
Persistent villous atrophy	6	112	1.73 (0.42–7.14)	0.45
40–59		433		
Mucosal healing	28	384	1.0	
Persistent villous atrophy	43	472	1.03 (0.63–1.68)	0.91
≥60		1509		
Mucosal healing	49	1464	1.0	
Persistent villous atrophy	67	1545	0.94 (0.64–1.36)	0.72
Calendar Year of Follow-up Biopsy				
1989 and before		319		
Mucosal healing	19	418	1.0	
Persistent villous atrophy	29	277	0.71 (0.39–1.29)	0.26
1990–1999		261		
Mucosal healing	36	213	1.0	
Persistent villous atrophy	64	299	1.15 (0.76–1.74)	0.50
2000 and after		210		
Mucosal healing	25	157	1.0	
Persistent villous atrophy	23	328	0.91 (0.51–1.61)	0.74

HR, Hazard ratio

IHD. Ischemic heart disease

†Adjusted for patient age at follow-up biopsy, gender, calendar period of follow-up biopsy, education, and duration of celiac disease at the time of follow-up biopsy.

**Table 4:** Risk of IHD type (myocardial infarction, stable angina, and unstable angina), and risk of death due to IHD in patients with CD who have persistent villous atrophy on follow-up biopsy, compared to those with mucosal healing.

	Number of events	Adjusted† HR (95% CI)	p value
IHD Overall			
Mucosal recovery	80	1.0	
Persistent villous atrophy	116	0.97 (0.73–1.30)	0.85
Angina pectoris			
Mucosal recovery	47	1.0	
Persistent villous atrophy	64	0.94 (0.64–1.38)	0.74
Unstable angina pectoris			
Mucosal recovery	14	1.0	
Persistent villous atrophy	16	0.71 (0.34–1.48)	0.36
Myocardial Infarction			
Mucosal recovery	70	1.0	
Persistent villous atrophy	106	1.03 (0.76–1.40)	0.85
Fatal MI			
Mucosal recovery	16	1.0	
Persistent villous atrophy	21	0.74 (0.38–1.43)	0.37

CD, celiac disease

HR, Hazard ratio

IHD. Ischemic heart disease

MI, Myocardial infarction

†Adjusted for patient age at follow-up biopsy, gender, calendar period of follow-up biopsy, education, and duration of celiac disease at the time of follow-up biopsy

### Persistent VA and prior IHD

Of 7,648 CD patients who underwent follow-up biopsy between 6 months and 5 years after initial CD diagnosis, 208 patients developed IHD prior to their follow-up biopsy. Although this was more common among those who developed persistent VA (3.6%) as compared to those with mucosal healing (2.1%), after adjusting for age, gender, calendar period of follow-up biopsy and educational attainment, there was no significant association between prior IHD and subsequent persistent VA (OR 1.07; 95%CI 0.79–1.46, p = 0.66).

### Risk of AF

Of 7,648 CD patients who underwent follow-up biopsy between 6 months and 5 years after their initial CD diagnosis, 118 patients developed AF prior to their follow-up biopsy. Of the remaining 7,530 patients, the incidence of AF was 266 per 100,000 person-years. Among those with persistent VA, the incidence of AF was 305 per 100,000 person-years, while among those with mucosal healing the incidence of AF was 226 per 100,000 person years. This yielded a positive unadjusted association between persistent VA and AF (HR 1.31; 95%CI 0.99–1.73, p = 0.06).

After adjusting for the above covariates, there was no significant difference in the risk of AF between those with mucosal healing and those with persistent VA (HR 0.98; 95%CI 0.74–1.30, p = 0.88). As was the case in IHD, the primary confounder was age; addition of age alone into the model led to a diminution of the HR from 1.31 to 1.03. The association between persistent VA and AF remained null when the analysis was repeated, using age as the time scale (HR 0.94; (95%CI 0.71–1.26, p = 0.69). Factors associated with an increased risk of AF included male sex (HR 1.98; 95%CI 1.49–2.62, p<0.0001), and increased age (HR per year 1.10;95% 1.09–1.11, p<0.0001). As was the case for IHD, adding diabetes to the multivariate model did not change the null association between persistent VA and AF (HR 0.98; 95%CI 0.74–1.30, p = 0.89). The association between diabetes and AF did not meet statistical significance (HR 1.43; 95%CI 0.75–2.71, p = 0.28). Quantifications of the association between persistent VA and AF stratified by gender, age, and calendar period are shown in Table 5. When restricting the population to adults (age ≥20 years), the incidence of AF was 543 per 100,000 person years of observation, and there was no association between persistent VA and risk of AF (HR 0.96; 95%CI 0.72–1.28, p = 0.78).

**Table 5:** Risk of atrial fibrillation in patients with CD according to follow-up histology.

Stratum	Number of events	Incidence per 100,000 PY	Adjusted† HR (95% CI)	p value
Overall		266		
Mucosal healing	85	226	1.0	
Persistent villous atrophy	120	305	0.98 (0.74–1.30)	0.88
Gender				
Male		389		
Mucosal healing	42	312	1.0	
Persistent villous atrophy	66	462	1.00 (0.67–1.48)	0.98
Female		197		
Mucosal healing	43	179	1.0	
Persistent villous atrophy	54	215	1.01 (0.67–1.52)	0.96
Age at follow-up biopsy (years)				
<20		5		
Mucosal healing	0	0		
Persistent villous atrophy	2	10	NC	NC
20–39		63		
Mucosal healing	6	82	1.0	
Persistent villous atrophy	2	37	0.34 (0.07–1.80)	0.21
40–59		341		
Mucosal healing	21	282	1.0	
Persistent villous atrophy	36	389	1.21 (0.69–2.12)	0.50
≥60		1718		
Mucosal healing	58	1695	1.0	
Persistent villous atrophy	80	1735	0.94 (0.67–1.32)	0.72
Calendar Year of Follow-up Biopsy				
1989 and before		198		
Mucosal healing	14	305	1.0	
Persistent villous atrophy	16	151	0.61 (0.29–1.26)	0.18
1990–1999		270		
Mucosal healing	42	247	1.0	
Persistent villous atrophy	62	287	0.87 (0.59–1.30)	0.51
2000 and after		306		
Mucosal healing	29	181	1.0	
Persistent villous atrophy	42	582	1.38 (0.85–2.23)	0.19

CD, celiac disease; HR, Hazard ratio; NC Not calculated.

†Adjusted for patient age at follow-up biopsy, gender, calendar period of follow-up biopsy, education, and duration of celiac disease at the time of follow-up biopsy

## Discussion

In this analysis of a population-based database of patients with CD who underwent a follow-up biopsy, we found that there was no independent increased risk of developing IHD or AF among patients with persistent VA compared to those with mucosal healing. This null finding was present in both genders, across age strata, and persisted over time after follow-up biopsy. These negative findings suggest that follow-up histology does not risk-stratify patients for the development of IHD or AF, with the caveat that a small positive association cannot be ruled out in certain subgroups due to limited statistical power.

We had hypothesized that mucosal healing would be associated with a decreased risk of subsequent IHD and AF. This hypothesis was based on the observation that patients with CD develop IHD and AF at a greater rate than would be expected in the general population[8] despite the observation that traditional IHD risk factors such as smoking and hyperlipidemia are less common in patients with CD.[28–30] It seemed plausible that the chronic inflammatory state inherent to CD was responsible for this increased, and that chronic inflammation reflected in follow-up histology could be of importance to assess cardiovascular risk in these patients. There is precedent for such a phenomenon, as patients with rheumatoid arthritis have an increased risk of cardiovascular events and this risk has been attributed to a chronic inflammatory state.[31] Moreover, chronic inflammation as reflected by the erythrocyte sedimentation rate appears to correlate with the severity of VA in patients with CD.[32] Our hypothesis was bolstered by the observation that the risk of IHD has previously been shown to be highest in the first year after diagnosis of CD (HR 1.77) with a diminished (but still significantly increased) risk beyond 5 years (HR 1.23), [8] suggesting that for a proportion of patients with CD who strictly adhere to the gluten-free diet, the histologic activity normalizes, and immune activation decreases along with cardiovascular risk.

Our null findings with regard to follow-up histology stand in contrast to this theoretical model. One potential explanation for these null findings is that VA is not an adequate marker for immune activation in patients with CD. Indeed, the previous study examining IHD risk in CD found that elevated risk of IHD in patients with CD (HR 1.19) was similar in magnitude to those with normal histology but elevated CD antibodies (HR 1.14), a phenomenon potentially representing future CD.[4] If such patients without VA are at increased risk of IHD, then healing of VA in CD may not be expected to impact IHD risk. Another possibility is that patients with persistent VA on a first follow-up biopsy may not reflect long-term mucosal healing rates; indeed, in one study of patients undergoing serial small intestinal biopsy at a referral center, the prevalence of mucosal healing was greater at 5 years (66%) than at 3 years (34%), suggesting that some patients gradually heal over subsequent years.[18] In this population-based database, the prevalence of persistent VA was similar among those whose follow-up biopsy was performed 1–2 years after diagnosis and 2–5 years after diagnosis, [27] suggesting that gradual mucosal healing is not a widespread phenomenon, at least within this time-frame. Another potential explanation for our null findings is that patients who underwent follow-up biopsy may represent a healthier subset of patients with CD than those who did not. Indeed, a follow-up biopsy may be deferred due to medical comorbidities which may in turn impact the risk of IHD. Supporting this notion is our finding, previously reported, that the overall mortality risk of patients who underwent follow-up biopsy was slightly lower than those CD patients who did not undergo follow-up biopsy.[20] Our null findings suggest that, although there is evidence that the risk of IHD along with intermediate biomarkers diminishes over time, [12] mucosal healing is not a marker for reduced cardiovascular risk. In our analysis of AF, another cardiac condition that is associated with CD, we likewise found that persistent VA is not an independent predictor of this outcome.

Although our adjusted risk estimate showed no significant association between persistent VA and the subsequent development of IHD, the unadjusted incidence of IHD was greater among those with persistent VA (298 per 100,000 person-years) compared to those with mucosal healing (214 per 100,000 person-years). An unadjusted Cox model found a greater risk of IHD among those with persistent VA that nearly met statistical significance (HR 1.30; 95% CI 0.97–1.73, p = 0.08). This contrast between unadjusted and adjusted risk estimates is driven primarily by the confounding influence of age. Increased age itself is associated with persistent VA, and this may be an intrinsic effect of aging, rather than a reflection of dietary adherence.[27] Increased age is associated with an increased risk of persistent VA, [27,33,34] and is a strong predictor of IHD.[35] Indeed, we found that when adjusting only for age, the risks of IHD and AF moved decisively towards the null. Therefore, although patients with persistent VA are more likely to develop IHD, this is primarily because they are older than those with mucosal healing. This phenomenon was also present in our analysis of AF, as age is associated with this outcome as well as with persistent VA.

This is the first study to our knowledge investigating the risk of IHD and AF according to follow-up histology in CD. Strengths of this study include its large sample size; despite the fact that the majority of patients in this database were children, the large sample size allowed us to test for this association in 1,063 patients older than 60 years, an age group in which IHD and AF are common.[36] We were also able to examine whether certain IHD subtypes (angina pectoris versus myocardial infarction) were more common in patients with persistent VA. Limitations of this study include the fact that data on only one follow-up biopsy were available, leaving open the possibility that a one-time biopsy result may not affect IHD risk but long term mucosal healing may still exert an effect. The low number of events in the younger age strata limited our ability to generate precise risk estimates for these groups, given the rarity of IHD and AF in individuals younger than 40. This also impacted the overall power of this study, and it remains possible that persistent VA exerts an influence on the outcomes of IHD and AF that our study was insufficiently powered to detect. As the increased risk of IHD in CD is modest in magnitude (with a previous study finding a HR of 1.19), [8] perhaps the association between follow-up histology and cardiovascular outcomes is similarly modest in magnitude. Indeed, this was the case in our prior study regarding lymphoproliferative malignancy, in which the overall risk of that outcome compared to the general population (HR 2.82) was greater than the risk-stratifying effect of persistent VA compared to CD patients who exhibited mucosal healing (HR 2.26).[21]

The reliance on claims codes for the diagnosis of CD leaves open the possibility of misclassification, but this concern is largely mitigated by a previous validation study demonstrating a high positive predictive value for the use of the claims codes employed in the development of this database.[23] Hospital-based diagnoses of MI and AF in particular have been shown to have high accuracy in Sweden, [37–39] although it should be admitted that IHD diagnoses were not adjudicated specifically for the purpose of this study. We lacked data regarding circulating markers of inflammation, such as C-reactive protein (CRP), which correlates with IHD risk.[40] Of note, a previous analysis of this database found that CRP levels among CD patients with IHD were similar to CRP levels to non-CD patients with IHD.[11]

We acknowledge that this study is limited by a lack of data regarding several important risk factors for IHD and for AF. Hypocholesterolemia is a feature of CD, [30] and increases in cholesterol levels, particularly high density lipoproteins, have been observed after commencement of the gluten-free diet.[41] In this population-based database of patients with CD who underwent follow-up biopsy, our ability to determine risk factors for both of these conditions was limited to ascertainment of age, gender, and diabetes. We therefore could not adjust for risk factors for IHD and/or AF, such as hypertension, dyslipidemia, smoking, alcohol use, and hyperthyroidism. It has been shown that traditional cardiovascular risk factors (such as smoking and hypertension) are less likely to be present in individuals with CD who had an ischemic cardiac event, as compared to non-CD patients who had an ischemic cardiac event.[11] While the risk for AF has been shown to be increased in patients with CD, a similar analysis of comparative risk factors has not been performed to date. Because we lacked data on these traditional risk factors, if they correlated positively with the outcomes of IHD and AF, and negatively with the exposure of persistent VA, negative confounding remains a possibility; adjustment for these variables may move our risk estimates away from the null. Patients with CD tend to have more favorable lipid profiles and may have lower rates of smoking than the general population, [30] though data on smoking are conflicting.[42] Of note, we found that diabetes exerted a modest risk on our outcomes, which did not meet statistical significance, and that adjusting for diabetes in our analysis did not affect the null association between persistent VA and these outcomes. Therefore, although the lack of information regarding smoking, dyslipidemia, and hypertension is a limitation of our study, it is unlikely that this limitation is unduly influencing our finding of a null association between persistent VA and the outcomes of IHD and AF.

In conclusion, this analysis of more than 7,000 patients with CD undergoing follow-up biopsy found no significant association between persistent VA and the subsequent development of IHD or AF. Although patients with persistent VA had higher rates of IHD and AF, this relationship was due to the confounding influence of age. As there was no protective effect of mucosal healing in decreasing the risk of IHD, our results suggest that clinical management of CD does not necessarily alter cardiovascular risk, and that other factors such as genetic influences may explain the relationship between CD and cardiovascular disease. Our results also suggest that even patients with mucosal healing require surveillance for IHD, coronary risk factor modification, and preventive measures such as aspirin in select patients. Future studies should investigate novel biomarkers of inflammation in CD that may impact the risk of IHD and AF.

## Supporting Information

S1 Table Risk of IHD among all patients with CD who had a follow-up biopsy between 6 months and 5 years after initial CD diagnosis, compared to their matched controls. (DOCX)
