# Supplementary material for: Mucosal Healing and the Risk of Ischemic Heart Disease or Atrial Fibrillation in Patients with Celiac Disease; A Population-Based Study
Source: PLoS One. 2015 Jan 30;10(1):e0117529. doi: 10.1371/journal.pone.0117529 (PMC4312018; doi:10.1371/journal.pone.0117529)
Supplement: S1 Table — (DOCX) [file pone.0117529.s001.docx]

**Supplementary Table 1:** Risk of IHD among all patients with CD who had a follow-up biopsy between 6 months and 5 years after initial CD diagnosis, compared to their matched controls.

| **Stratum** | **Adjusted†**  **HR (95% CI)** | **p value** |
| --- | --- | --- |
| **Overall** |  |  |
| Celiac Disease | 1.09 (0.95-1.25) | 0.22 |
| Controls  Persistent villous atrophy  Controls  Mucosal healing  Controls | 1.0  1.07 (0.90-1.29)  1.0  1.11 (0.90-1.38)  1.0 | 0.43  0.33 |
| **Gender** |  |  |
| Male |  |  |
| Celiac Disease | 1.21 (0.99-1.47) | 0.06 |
| Controls | 1.0 |  |
| Female |  |  |
| Celiac Disease | 0.99 (0.82-1.20) | 0.93 |
| Controls | 1.0 |  |
| **Age at diagnosis** |  |  |
| <20 |  |  |
| Celiac Disease | NC | NC |
| Controls |  |  |
| 20-39 |  |  |
| Celiac Disease | 1.59 (0.90-2.82) | 0.11 |
| Controls | 1.0 |  |
| 40-59 |  |  |
| Celiac Disease | 1.02 (0.81-1.27) | 0.89 |
| Controls | 1.0 |  |
| ≥60 |  |  |
| Celiac Disease | 1.09 (0.91-1.31) | 0.34 |
| Controls | 1.0 |  |

CD, celiac disease

HR, Hazard ratio

IHD. Ischemic heart disease

NC, Not calculated due to insufficient number of events in individuals younger than 20 (1 in CD group, 3 in controls)

**†**Controls were matched to CD patients by age, gender, and calendar period, and HR’s included adjustment for educational attainment.
